# Pressure‐Induced Metallization of Lead‐Free Halide Double Perovskite (NH_4_)_2_PtI_6_

**DOI:** 10.1002/advs.202203442

**Published:** 2022-08-15

**Authors:** Jiaxiang Wang, Lingrui Wang, Yuqiang Li, Ruijing Fu, Youjia Feng, Duanhua Chang, Yifang Yuan, Han Gao, Sheng Jiang, Fei Wang, Er‐jia Guo, Jinguang Cheng, Kai Wang, Haizhong Guo, Bo Zou

**Affiliations:** ^1^ Key Laboratory of Material Physics Ministry of Education School of Physics and Microelectronics Zhengzhou University Zhengzhou 450052 P. R. China; ^2^ Tianjin Key Laboratory of Optoelectronic Detection Technology and Systems School of Electrical and Electronic Engineering Tiangong University Tianjin 300387 P. R. China; ^3^ School of Applied Physics and Materials Wuyi University Jiangmen 529020 P. R. China; ^4^ Shanghai Advanced Research Institute Chinese Academy of Sciences Shanghai 201210 P. R. China; ^5^ Beijing National Laboratory for Condensed Matter Physics and Institute of Physics Chinese Academy of Sciences Beijing 100190 P. R. China; ^6^ State Key Laboratory of Superhard Materials College of Physics Jilin University Changchun 130012 P. R. China

**Keywords:** high‐pressure, metallization, perovskites, phase transition, Shockley–Queisser limit

## Abstract

Metallization has recently garnered significant interest due to its ability to greatly facilitate chemical reactions and dramatically change the properties of materials. Materials displaying metallization under low pressure are highly desired for understanding their potential properties. In this work, the effects of the pressure on the structural and electronic properties of lead‐free halide double perovskite (NH_4_)_2_PtI_6_ are investigated systematically. Remarkably, an unprecedented bandgap narrowing down to the Shockley–Queisser limit is observed at a very low pressure of 0.12 GPa, showing great promise in optoelectronic applications. More interestingly, the metallization of (NH_4_)_2_PtI_6_ is initiated at 14.2 GPa, the lowest metallization pressure ever reported in halide perovskites, which is related to the continuous increase in the overlap between the valence and conduction band of I 5p orbital. Its structural evolution upon compression before the metallic transition is also tracked, from cubic *Fm‐*3*m* to tetragonal *P*4/*mnc* and then to monoclinic *C*2/*c* phase, which is mainly associated with the rotation and distortions within the [PtI_6_]^2–^ octahedra. These findings represent a significant step toward revealing the structure–property relationships of (NH_4_)_2_PtI_6_, and also prove that high‐pressure technique is an efficient tool to design and realize superior optoelectronic materials.

## Graphical abstract

An unprecedented bandgap narrowing of lead‐free halide double perovskite (NH_4_)_2_PtI_6_ down to the Shockley–Queisser limit is observed at a very low pressure of 0.12 GPa. More importantly, the lowest metallization pressure (14.2 GPa) is achieved ever reported in halide perovskites.

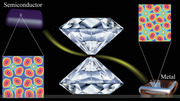

## 1 Introduction

Lead‐free halide double perovskites (HDPs) have emerged as effective and green alternatives to the well‐studied lead halide perovskites (LHPs) due to their easy processability, high stability, and reduced toxicity.^[^ ^1^ ^]^ The HDPs structure is designed with either A_2_B(IV)X_6_ or A_2_B(I)B(III)X_6_ chemical generalizations based on the heterovalent substitution strategy, that is, a pair of Pb(II) is substituted by a tetravalent B(IV) cation to form a unique network structure with [BX_6_]^2–^ independent octahedral units spaced apart in a vacancy‐ordered arrangement; or by a monovalent B(I) cation and a trivalent B(III) cation to form a 3D structure with alternating angular sharing. Due to the lack of the connectivity in the [BX_6_]^2–^ units, the A_2_BX_6_ HDPs exhibit different structural, optical, and optoelectronic properties in comparison to the ABX_3_ LHPs.^[^ ^2^ ^]^ In particular, the A_2_BX_6_ HDPs have shorter while stronger bonds of the B—X bonds within the [BX_6_]^2–^ units compared to that of the ABX_3_ LHPs, resulting in their better stability against moisture or water.^[^ ^3^ ^]^ On the other hand, the shorter and stronger B—X bonds within the [BX_6_]^2–^ octahedron greatly affect the electronic structure of the A_2_BX_6_ HDPs, leading to an increased overlap of the electron cloud with a smaller bandgap.^[^ ^2^, ^3^ ^]^ Thus, features of HDPs motivate further study of these materials in an effort to elucidate the structure–property relationships and improve their potential applications.

Perovskite materials with small bandgaps are required to harvest a broader range of the solar spectrum. Pressure is a green, facile, and effective tool to tune the band structure of the perovskite materials without inducing the chemical impurities.^[^ ^4^ ^]^ To date, high pressure has been applied on perovskites to optimize their physical properties and induce novel phenomena such as pressure‐induced emission enhancement,^[^ ^5^ ^]^ superconductivity, piezochromism, etc.^[^ ^6^ ^]^ Recently, Xu et al. have found that the bandgap of *α*‐FAPbI_3_ decreases and reaches the Shockley–Queisser optimum bandgap (1.34 eV) during compression in relatively low pressure regimes (below 2.1 GPa).^[^ ^7^ ^]^ The pressure‐realized bandgap requirement by the Shockley–Queisser limit were also observed in Cs_3_Bi_2_I_9_ (12.1 GPa), (CH_3_NH_3_)_3_Bi_2_I_9_ (13.2 GPa), CsPbI_3_ (15.0 GPa), Cs_3_Sb_2_I_9_ (20.1 GPa), etc.^[^ ^4^, ^8^ ^]^ When the pressure is increased, pressure‐induced closure of the 2.5‐eV‐bandgap in CsPbI_3_ at a pressure occurs above 39.3 GPa.^[^ ^8c^ ^]^ Similar phenomena of the pressure‐induced metallization in Cs_3_Bi_2_I_9_ (28.0 GPa), Cs_3_Sb_2_I_9_ (44.3 GPa), MAPbI_3_ (60.0 GPa), and other halide perovskites have also been observed.^[^ ^6a^ ^]^ From the view of the practical application, the pressure required to change the physical properties of the material needs to be as low as possible. In addition to the above reports, there still leaves room for decreasing the critical pressure reaching the Shockley–Queisser limit and metallization in the perovskite materials. The HDPs‐based (NH_4_)_2_PtI_6_ possesses a bandgap of 1.36 eV under ambient conditions, extremely close to the Shockley–Queisser limit, and appropriate schemes are needed to explore its potential properties and effectively expand the application of this material.

Herein, we performed a systematic high‐pressure study to explore the structural, electronic, and optical properties of the HDPs‐based (NH_4_)_2_PtI_6_ by the means of in situ angle‐dispersive synchrotron X‐ray diffraction (ADXRD), Raman, UV−vis absorption, temperature‐dependent electrical resistivity experiments, as well as density functional theory (DFT) calculations. The bandgap of (NH_4_)_2_PtI_6_ reaches the Shockley–Queisser optimum bandgap as the pressure increases to very low pressure of 0.12 GPa. And then (NH_4_)_2_PtI_6_ exhibits persistent band‐gap narrowing, accompanied by two phase transitions from cubic to tetragonal and to monoclinic phase. Surprisingly, above 14.2 GPa, the transport property of (NH_4_)_2_PtI_6_ exhibits a semiconductor‐to‐metal transition, and to our best knowledge, this metallization pressure is the lowest to date ever reported in halide perovskites. Our work demonstrates that the bandgap of (NH_4_)_2_PtI_6_ can be fine‐tuned by the pressure and motivates further exploration of the high‐pressure behaviors of more HDPs.

## 2 Results and Discussion

**Figure 1:**
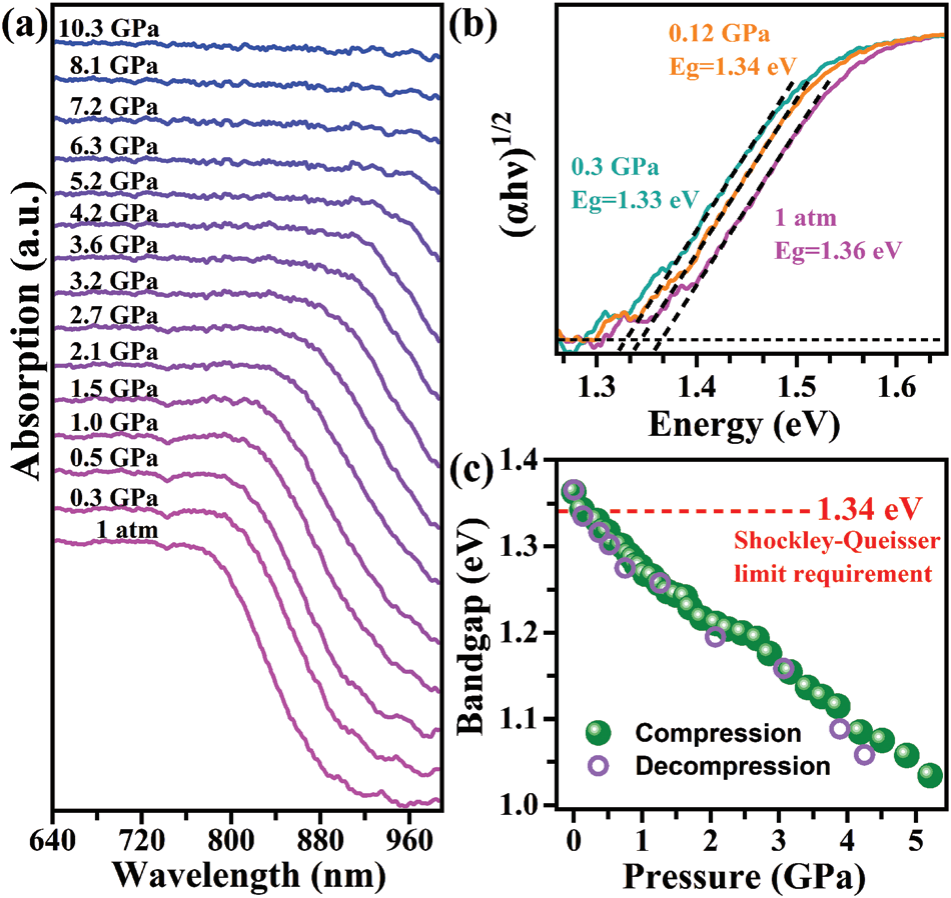
a) In situ evolution of the absorption spectra of (NH_4_)_2_PtI_6_ with the pressure. b) The indirect bandgap Tauc plots under 1 atm, 0.12 GPa, and 0.3 GPa. c) The bandgap evolutions of (NH_4_)_2_PtI_6_ as a function of the pressure. The symbol size covers the size of the error bars.

As (NH_4_)_2_PtI_6_ exhibits significant promise as an absorber in solar cells due to its near‐ideal bandgap for solar harvesting, there is a strong incentive to investigate the bandgap behaviors under the pressure. **Figure** **1** depicts the pressure‐induced variations in the absorbance properties and bandgap of (NH_4_)_2_PtI_6_. As seen in Figure 1a, a distinct absorption edge can be visible at around 945.00 nm. The absorption edge gradually redshifts as the pressure increases, tails into the near‐infrared at 1.0 GPa and extends into the near‐infrared above 7.2 GPa, indicating the pressure can modify the optical property of (NH_4_)_2_PtI_6_ toward enhanced absorption in the visible and infrared region. After decompression, the absorption spectrum almost returns to its initial state (Figure S1, Supporting Information). The evolution of the measured bandgaps of (NH_4_)_2_PtI_6_ can be effectively evaluated according to the shift of the absorption edge by Tauc plots (Figures 1b,c).^[^ ^9^ ^]^ The bandgap of (NH_4_)_2_PtI_6_ is about 1.36 eV at ambient pressure and continuously decreases with increasing the pressure. It should be noted that the bandgap is reduced to ≈1.34 eV at 0.12 GPa, achieving the Shockley–Queisser optimum bandgap (1.34 eV) where the maximum theoretical energy conversion efficiency (33%) of solar cells can obtain.^[^ ^10^ ^]^ Moreover, this is the lowest pressure (0.12 GPa) that the bandgaps of the halide perovskites ever reported that can reach the Shockley–Queisser optimum bandgap (as summarized in Table S1, Supporting Information). Bandgap determination becomes difficult above 5.2 GPa because the absorption spectra extend into the near‐infrared region.

**Figure 2:**
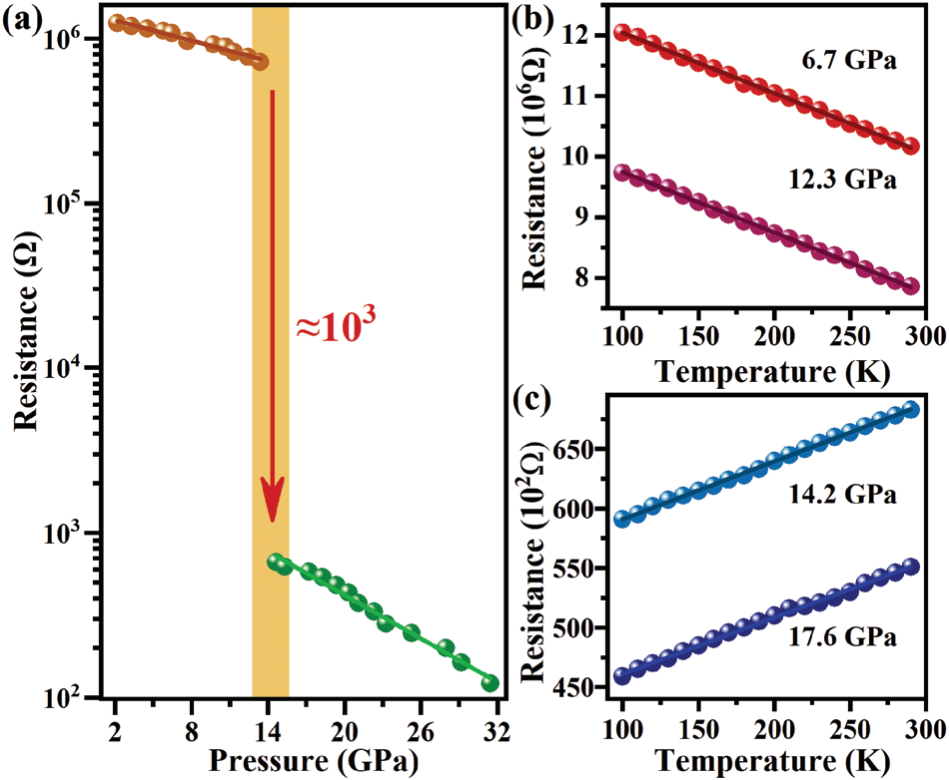
a) Room‐temperature resistance of (NH_4_)_2_PtI_6_ as a function of pressure. b,c) The pressure dependence of resistance in (NH_4_)_2_PtI_6_ as a function of temperature.

Considering the trend of the bandgap of (NH_4_)_2_PtI_6_ exhibits persistent narrowing under the pressure, it is predicted that the bandgap closure will emerge when sufficient compression pressure is applied. In this regard, temperature‐dependent resistance up to 31.4 GPa was measured to determine if (NH_4_)_2_PtI_6_ has undergone a semiconducting‐metal transition. The room‐temperature resistance of (NH_4_)_2_PtI_6_ as a function of the pressure is shown in **Figure** **2**a. At ambient conditions, (NH_4_)_2_PtI_6_ shows poor conductivity due to the disconnected nature of the isolated inorganic skeleton structure. Upon compression, the resistance of (NH_4_)_2_PtI_6_ exhibits an exponential decrease with the pressure. Below 13.4 GPa, the decrease of the resistivity is fit by log(*R*) = 6.1(5)−0.02(0)*P*, slower than that above 14.6 GPa, fit by log(*R*) = 3.5(1)−0.04(3)*P*, resistivity (*R*) in unit of Ω cm^−1^ and the pressure (*P*) in unit of GPa. Surprisingly, the electrical resistance of (NH_4_)_2_PtI_6_ decreases more than three orders of magnitude when the pressure is above 13.4 GPa, indicating an electronic structure transition. The evolution of the resistance under the different pressures was subsequently investigated in the temperature range of 100.0–290.0 K, as shown in Figure 2b,c. The resistance of (NH_4_)_2_PtI_6_ under four different pressures all shows the linear variation with the temperature over the test interval. The temperature‐dependent resistance (*R*–*T*) demonstrates a negative d*R*/d*T* at 6.7 GPa and 12.3 GPa, implying that (NH_4_)_2_PtI_6_ is at semiconductor state with thermally activated carriers. However, the positive d*R*/d*T* can be observed above 14.2 GPa, which is a hallmark of the metallic character. In other words, the *R*–*T* curves of (NH_4_)_2_PtI_6_ confirm a semiconductor–metal transition at 14.2 GPa, the lowest metallization pressure ever reported so far in the halide perovskites as summarized in Table S2, Supporting Information. As discussed above, it can be seen that the optical and electrical property of (NH_4_)_2_PtI_6_ can be significantly modulated by applying a moderate pressure.

**Figure 3:**
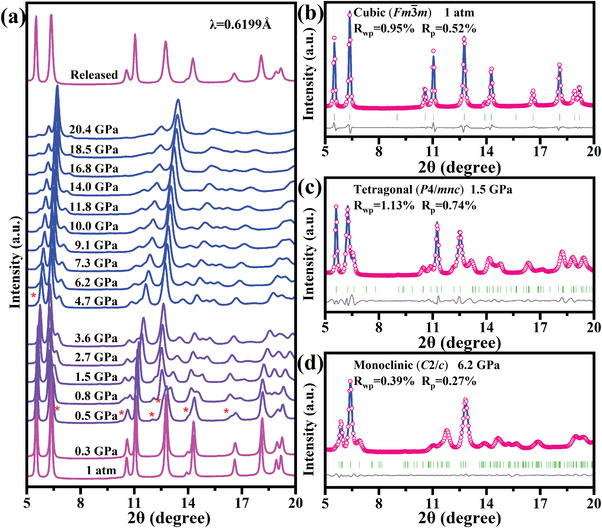
a) High‐pressure evolution of ADXRD patterns of (NH_4_)_2_PtI_6_ as a function of the pressure. Red asterisks mark the appearance of new diffraction peaks. b–d) Rietveld refinements of (NH_4_)_2_PtI_6_ crystal at ambient pressure (cubic phase), 1.5 GPa (tetragonal phase), and 6.2 GPa (monoclinic), respectively.

In situ synchrotron pressure‐dependent structural characterization was used to investigate the mechanisms behind the bandgap evolution and to derive the structure–property relationships. **Figure** **3**a shows the typical in situ ADXRD patterns of (NH_4_)_2_PtI_6_ upon the compression to 20.4 GPa and after complete decompression. (NH_4_)_2_PtI_6_ crystallizes into a cubic symmetry under ambient pressure, which is consistent with the previous reports and confirms its high phase purity.^[^ ^11^ ^]^ As the pressure increases, the Bragg diffraction peaks move to higher angles, indicating the contraction of the unit cell. When the pressure is increased to 0.5 GPa, new Bragg diffraction peaks appear in the XRD patterns, marked with asterisks in Figure 3a. These changes indicate that the cubic phase of the (NH_4_)_2_PtI_6_ crystal undergoes a symmetry‐lowering phase transition. Upon further compression to 4.7 GPa, accompanied by the shift and broadening of the original peaks, a new peak suddenly appears at about angle 5.5, indicating the second structural phase transition occurs. The high‐pressure phase of the secondary transformation sustains up to 20.4 GPa. When combined with the results of the high‐pressure electrical experiments, the semiconductor–metal transition of (NH_4_)_2_PtI_6_ is most likely induced by the formation of a new ordered metal phase under the high pressure rather than by the amorphization. However, the XRD pattern shows no drastic change in the crystal structure near the transition pressure of in the crystal structure near the transition pressure of 14.0 GPa, therefore the sudden change of the resistance of (NH_4_)_2_PtI_6_ could be ascribed to the dramatic change in the electronic structure. Upon decompression, the sample gradually reverts to its original (NH_4_)_2_PtI_6_ crystal structure under ambient conditions, indicating that the structural change is reversible. Also, this reversibility suggests that the metallization is probably not the result of irreversible material decomposition. The detailed variation of the crystal structure of (NH_4_)_2_PtI_6_ with the pressure was determined by the Rietveld refinement method (Figure 3b–d, Figure S2, and Tables S3,S4, Supporting Information).^[^ ^12^ ^]^ Under the initial conditions, (NH_4_)_2_PtI_6_ possesses a cubic structure with the space group *Fm‐*3*m* and the lattice parameters *a* = *b* = *c* = 11.5(8) Å and *V* = 1389.1(8) Å. The 1.5 GPa high‐pressure phase refinement forms a tetragonal structure with space group *P*4/*mnc*, which is obtained by rotating the [PtI_6_]^2–^ regular octahedral in the *ab* plane along the *c*‐axis direction. The lattice parameters are defined as follows: *a* = 7.6(4) Å, *b* = 7.6(4) Å, and *c* = 11.6(6) Å. During the cubic to tetragonal phase transition, the parameters *a* and *b* in the *ab* plane appear to contract abruptly, while the parameter *c* increases slightly and then decreases gradually. That is, the unit cell is compressed in the *ab* plane, while it is first slightly stretched and then gradually compressed in the *c*‐axis direction. Meanwhile, the rotation angle of the octahedra in the tetragonal phase is about 35.2 degrees compared to the cubic phase. That is mostly related to the shrinkage and rotation of [PtI_6_]^2–^ octahedra. At 6.2 GPa, the monoclinic cell with the space group *C*2/*c* is used to fit the split diffraction peaks. The lattice parameters are: *a* = 7.1(4) Å, *b* = 7.4(1) Å, *c* = 11.0(9) Å, and *β* = 89.3(1)°. The I—Pb—I bond angle along the *c*‐axis direction deviates from the previous 180°, the I—Pb—I bond angle in the *ab* plane changes from 90.00° to 88.03°, and tilt deformation occurs within the [PtI_6_]^2–^ octahedra. This implies that the second structural phase transition is mainly due to the distortions within the [PtI_6_]^2–^ octahedra. These two transitions are both of first order type due to the significant discontinuities in the lattice parameters (Table S5, Supporting Information). Fitting with the third order Birch–Murnaghan equation of state, the bulk moduli *B*_0_ = 24.5(1) GPa for the tetragonal phase, and *B*_0_ = 33.6(4) GPa for the monoclinic phase. The relatively higher *B*_0_ presents the less compressible nature of monoclinic phase. The energy differences among these three structures (cubic (*Fm*‐3*m*), tetragonal (*P*4/*mnc*), and monoclinic (*C*2/*c*)) are calculated in Figure S3, Supporting Information, suggesting a relative stability of monoclinic under the pressure.

**Figure 4:**
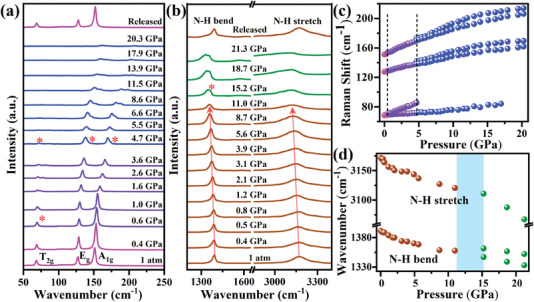
a,b) Representative Raman and IR spectra of (NH_4_)_2_PtI_6_ and c,d) the corresponding frequency shifts as a function of pressure.

We also performed in situ high‐pressure Raman and infrared (IR) experiments to investigate the effect of the variation of the inorganic octahedra on the local lattice vibration of (NH_4_)_2_PtI_6_ and the behavior of the NH_4_^+^ cations during the compression. **Figure** **4**a,c shows the representative Raman spectra of (NH_4_)_2_PtI_6_ and the corresponding frequency shifts as a function of the pressure. We observed three Raman vibrational modes in the low‐wavenumber range of 50.0–250.0 cm^–1^ at ambient conditions, which corresponds to the lattice modes associated with the Pt—I vibrational modes consisting of the ionic interactions. Based on the allocation of analogues in previous studies, the band at 67.0 cm^–1^ is ascribed to the T_2g_ triply degenerate bending vibration and has the lowest frequency of three, while the two bands at 128.0 and 151.0 cm^–1^ belong to the E_g_ doubly degenerate stretching vibration and A_1g_ symmetric stretching vibration, respectively.^[^ ^13^ ^]^ As the pressure increases, all modes shift to higher frequency, which is consistent with the contraction of the [PtI_6_]^2–^ octahedra. When the pressure increases to 0.6 GPa, the splitting of the bending vibration of the Pt—I bonds with the T_2g_ symmetry indicates the appearance of the first phase transition associated with the inorganic octahedral rotation. Under 4.7 GPa, the E_g_ and A_1g_ modes split and the T_2g_ mode starts to broaden, indicating that the distortion has occurred within the [PtI_6_]^2–^ octahedron. The vibration modes completely disappear under 20.3 GPa and return to their initial state after complete release of the pressure. The behavior of the NH_4_^+^ cation upon compression can be understood by in situ high‐pressure IR spectroscopy experiments (Figure 4b,d). The N—H bending and N—H stretching are simultaneously red‐shifted upon the pressure. Since H in the NH_4_^+^ cation is more positively charged, this may be a result of the enhanced electrostatic interaction between H and I, and leading to an increase in the N—H bond length and a weakening of the bend and tensile vibrational frequencies.^[^ ^14^ ^]^ Under 15.2 GPa, a new vibrational peak (marked with an asterisk) appears in the N—H bending vibrational mode, indicating drastic change in the environment around the NH_4_^+^ cation. This is consistent with a semiconductor‐metal transition under the pressure. Therefore, a significant role of the hydrogen bonding in the compressed systems can be anticipated.^[^ ^15^ ^]^

**Figure 5:**
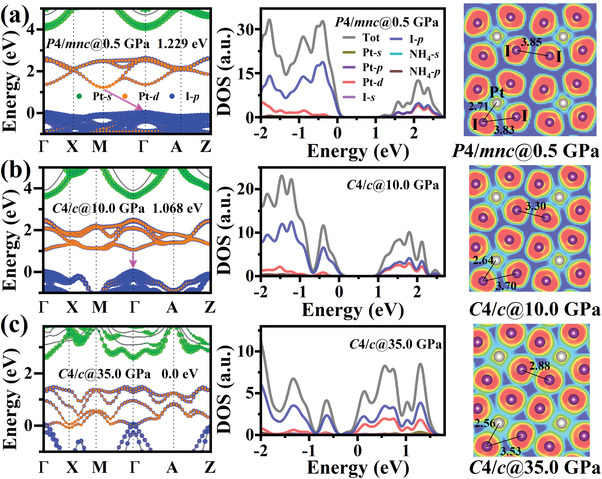
The projected band structures, DOS, and 2D electron localization function (ELF) maps on the (001) plane cutting through the Pt and I atoms of (NH_4_)_2_PtI_6_ at a) 0.5 GPa (*P*4/*mnc*), b) 10.0 GPa (*C*4/*c*), and c) 35.0 GPa (*C*4/*c*), respectively. The data show the bond lengths of Pt—I and I—I in unit of Å.

Considering the well‐known issue of the underestimation of the bandgap in plain DFT calculations, all of the bandgap curves and band structure are calculated by the HSE06 method. The first‐principles DFT‐HSE06 calculated pressure‐dependent bandgap of (NH_4_)_2_PtI_6_ are shown in **Figure** **5** and Figure S4, Supporting Information. The calculated bandgaps exhibit a persistent narrowing with the increase of the pressure, which are consistent with the experimental results. And it can be roughly seen that the band edge mainly comes from the Pt 5d and I 5p orbitals. Meanwhile, the flexible organic cations [NH_4_]^+^ provide one positive charge and serve as the templates in octahedral frameworks but makes little contribution to the electronic properties. Under the pressure of 0.5 GPa, (NH_4_)_2_PtI_6_ undergoes a symmetry‐lowering phase transition from the cubic *Fm*‐3*m* to the tetragonal *P*4/*mnc*. The primitive cell of (NH_4_)_2_PtI_6_ changes from one formula in the cubic phase to double in the tetragonal phase. In other words, in Figure 5a, there are more energy levels, and the high symmetry point of *M* folded to *Γ* point. The (NH_4_)_2_PtI_6_ tetragonal phase crystal shows an indirect bandgap (from *M*(0.5, 0.5, 0) to *Γ*(0, 0, 0)) at the same time the bandgap changes to 1.23 eV. With the pressure continuing to increase above 5.0 GPa, the (NH_4_)_2_PtI_6_ crystal changes from the tetragonal to monoclinic phase with more rotation and distortion of the [PtI_6_]^2–^ octahedral (Table S2, Supporting Information). The bandgap narrows further and changes to a direct bandgap. As the pressure is increased to 10.0 GPa, the (NH_4_)_2_PtI_6_ crystal shows a direct bandgap with the bandgap energy of 1.07 eV (Figure 5b). With the pressure up to 35.0 GPa, the overlap between the I 5p and Pt 5d orbital shows the closure of the bandgap and metallic properties, as in Figure 5c. The material's properties change from the indirect to direct and metallic when the pressure is applied. This pressure‐induced gradual metallization has been explained by the continuous increase in the band overlap between the valence and conduction band. It can be seen that the electrons between Pt—I and I—I overlap more as the pressure increases, which is shown in the corresponding calculated electron localization function (ELF) of (NH_4_)_2_PtI_6_. As the pressure increases from 0.5 to 10.0 to 30.0 GPa, the Pt—I bond length changes from 2.71 to 2.64 to 2.56 Å, the intra‐octahedral I—I bond changes from 3.83 to 3.75 to 3.53 Å and the inter‐octahedral I—I bond changes from 3.85, 3.30 to 2.88 Å. It demonstrates that the inter‐octahedral bonds changes much faster than the intra‐octahedral I—I bonds. As a result, the shortest I—I bonds of (NH_4_)_2_PtI_6_ will shift from the intra‐ to inter‐octahedra as the pressure increases. The mechanism of this bond length change can be readily understood from the viewpoint of the stiffness of the octahedral and is similar to the HDPs‐based (NH_4_)_2_SeBr_6_.^[^ ^4^, ^16^ ^]^ As mentioned above, it can be seen that the bandgap narrowing and close are mainly caused by the overlapping evolution of the I 5p orbital. To confirm this assumption, we studied the bandgap evolution of the orthorhombic I bulk phase (*Cmca*) under the pressure. We found that the orthorhombic bulk I shows an indirect bandgap of 1.58 eV (with I—I bond of 2.81 and 3.34 Å) and close its bandgap at about 16.6 GPa (with the I—I bond of 2.99 and 2.99 Å) in HSE06 calculations (Figure S5, Supporting Information).^[^ ^17^ ^]^ We note that the I—I bond length of 2.99 Å in its bulk phase is even larger than the inter‐octahedral bond length of 2.88 Å (35.0 GPa) in (NH_4_)_2_PtI_6_ when they become metallic. In this respect, the HSE06 calculated metallic pressure for (NH_4_)_2_PtI_6_ is 35.0 GPa, larger than our experimental 14.2 GPa. This diversity has also been found in CsPbI_3_, which exhibits a metal transition under 39.3 GPa in the experiment but 50.0 GPa in DFT theoretical calculation.^[^ ^8c^ ^]^ By comparing the metallization process of the bulk phase iodine, we found that the contribution of the 5d orbitals of Pt to CBM gradually increases during the semiconductor‐metal phase transition of (NH_4_)_2_PtI_6_, and the broadening of the energy band indicates a decrease in the effective mass and an increase in the non‐localization of the electrons. These findings provide evidence to reveal the mechanism of the pressure‐induced semiconductor–metal phase transition with the crystal structure phase transition leading to electronic structure transformation, and also offers an effective strategy to design ordered perovskite metal phases obtained under lower pressures.

## 3 Conclusions

In summary, we comprehensively investigated the bandgap and structural evolutions of HDPs‐based (NH_4_)_2_PtI_6_ under the high pressure by combining the experimental measurements and theoretical calculations. The results show that the bandgap of (NH_4_)_2_PtI_6_ breaks through the Shockley–Queisser limit under a very low pressure of 0.12 GPa and exhibits closure under the pressure of about 14.2 GPa that related to the continuous increase in the overlap between the valence and conduction band of I 5p orbital. Meanwhile, the pressure‐induced structural evolutions of (NH_4_)_2_PtI_6_ originate from the cubic *Fm‐*3*m* to the tetragonal *P*4/*mnc* and then to the monoclinic *C*2/*c* phase associated with the rotation and distortions within the [PtI_6_]^2–^ octahedra. The analyzed exceptional photovoltaic and other optoelectronic properties motivate further exploration of this exceptionally versatile family of materials.

## Conflict of Interest

The authors declare no conflict of interest.

## Supporting information

Supporting Information

## Data Availability

The data that support the findings of this study are available from the corresponding author upon reasonable request.
