# Supplementary material for: Pressure‐Induced Metallization of Lead‐Free Halide Double Perovskite (NH4)2PtI6
Source: Adv Sci (Weinh). 2022 Aug 15;9(28):2203442. doi: 10.1002/advs.202203442 (PMC9534948; doi:10.1002/advs.202203442)
Supplement: Supplementary file 1 — Supporting Information [file ADVS-9-2203442-s001.pdf]

ADVANCED SCIENCE
Open Access

Supporting Information

for *Adv. Sci.*, DOI 10.1002/advs.202203442

Pressure-Induced Metallization of Lead-Free Halide Double Perovskite $(NH_4)_2PtI_6$

Jiaxiang Wang, Lingrui Wang, Yuqiang Li, Ruijing Fu, Youjia Feng, Duanhua Chang, Yifang Yuan, Han Gao, Sheng Jiang, Fei Wang, Er-jia Guo, Jinguang Cheng, Kai Wang*, Haizhong Guo* and Bo Zou*

Supporting Information

Pressure-Induced Metallization of Lead-Free Halide Double Perovskite $(NH_4)_2PtI_6$

Jiaxiang Wang,[#] Lingrui Wang,[#] Yuqiang Li, Ruijing Fu, Youjia Feng, Duanhua Chang Yifang Yuan, Han Gao, Sheng Jiang, Fei Wang,[*] Er-jia Guo, Jinguang Cheng, Kai Wang,[*] Haizhong Guo[*] and Bo Zou

*Corresponding author. Email: wfei@zzu.edu.cn; kaiwang@jlu.edu.cn; hguo@zzu.edu.cn

Table of Contents

Experimental Section

Theoretical calculation

Table S1. The bandgap at ambient conditions and the pressures reaching the S-Q limit bandgap of the halide perovskites ever reported.

Table S2. The metallization pressures of the halide perovskites ever reported.

Table S3. Refinement lattice parameters and refinement statistics at ambient pressure, 1.5 GPa, and 6.2 GPa for the $(NH_4)_2PtI_6$ crystal.

Table S4. The lattice parameters and unit cell volume of $(NH_4)_2PtI_6$ at different pressures.

Table S5. Pt-I bond lengths and I-Pt-I bond angles of $(NH_4)_2PtI_6$ under various pressures. The illustration shows the positions of atoms.

Figure S1. The absorption spectra of the $(NH_4)_2PtI_6$ crystal before and after the high-pressure treatments.

Figure S2. Schematic illustrations with respect to polyhedral views of the crystal structures of $(NH_4)_2PtI_6$ under high pressure.

Figure S3. The calculated energy differences per unit volume of difference phases under the pressure.

Figure S4. a) DFT-HSE06 calculated bandgap of $(NH_4)_2PtI_6$ under various pressures. The green-star shows the bandgap of cubic phase at ambient pressure. b) The projected band structure for the Pt and I atoms under ambient conditions. c) The calculated square of the transition dipole moment (P^2) along the high symmetry lines for VBM and VB1. d) Total and projected density of states (DOS and PDOS) in cubic phase under 1 atm of $(NH_4)_2PtI_6$.

Figure S5. The projected band structures of bulk I (*Cmca*) at a) 1 atm, b)16.0 GPa, respectively.

References

Experimental section.

Sample synthesis and high-pressure generation. A classical method was used to synthesize $(NH_4)_2PtI_6$. The symmetry-type diamond anvil cell (DAC) was adopted to generate high pressure. Considering the spectral properties, IIa-type diamonds with 400μm culet-size and high transmittance in the UV region were used for high-pressure optical experiments. A T301 stainless steel spacer with a pre-indented thickness of 42 μm was used and a 150-μm-diameter hole was drilled in the center of the indentation for the sample chamber. The pressure calibration was carried out using the ruby fluorescence method, and the use of silicone oil as a pressure transfer medium during compression. Silicon oil was utilized as the pressure transmitting medium (PTM) for optical absorption and XRD experiments, while the argon and KBr were employed as PTM for Raman and IR measurements.

***In situ* high-pressure experiments.** *In situ* high-pressure absorption spectra were performed using a fiber spectrometer (QE65000, Ocean Optics). *In situ* high-pressure Raman spectra were recorded using a spectrometer equipped with the liquid nitrogen cooled CCD (iHR550, Symphony Ⅱ, Horiba Jobin Yvon). The 532-nm-diode laser was used to excite the sample and the output power was 10 mW. *In situ* high pressure synchrotron ADXRD experiments were conducted at the BL15U1 beamline of Shanghai and 4W2 High Pressure Station of Beijing Synchrotron Radiation Facility. The experiments were carried out using a monochromatic X-ray beam with a wavelength of 0.6199 Å. The refinement of the XRD pattern was completed using the Reflex module in the Materials Studio software. The Pawley profile-fitting routine was first used to refine the cell parameters and search the space group, and then the Rietveld refinement was executed to obtain the crystal structural parameters. *In situ* electrical resistance experiments were performed using a Solartron 1260A impedance/gain phase analyzer with Solartron 1296A. High-pressure *in situ* electrical experiments were performed by film microcircuits, which are integrated on the diamond anvil surface by photolithography.

Theoretical calculation.

All the first-principles density functional theory (DFT) calculations are performed using the plane-wave pseudopotential as implemented in the Vienna ab-initial simulation package (VASP) code.[1] The electron-core interactions are described with the frozen-core projector-augmented wave pseudopotentials as parameterization by the Perdew-Burke-Ernzerhof (PBE) as the

exchange-correlation function with $5d^{10}5p^{6}5s^{2}$(Pt), $5p^{5}5s^{2}$(I), $2s^{2}2p^{3}$(N), and $1s^{1}$(H) treated explicitly as valence electrons.[2] A cut-off energy of 400 eV is used for all of the calculations. The convergence criteria, force on each atom converge to 0.01 eV/Å and total energy 1.0×10^{-5} eV, has been choose to optimizing the geometric structure. The vdW interaction is considered by using the DFT-D2 method of Grimme.[3] The DFT PBE method is used to obtain total energy, energy difference, and pressure trend.[4-6] The Heyd-Scuseria-Ernzerhof (HSE06) method is used to get the bandgap values and electronic structure.[7] VASPKIT, an interface for VASP calculation, has been used in data processing.[8]

**Halide Perovskite**	**Bandgap (1 atm)**	**S-Q limit GPa**	**Refs.**
**$(NH_4)_2PtI_6$**	1.36 eV	0.12	This work
**$Cs_3Bi_2I_9$**	2.06 eV	12.1	[9]
**$Cs_3Sb_2I_9$**	2.34 eV	20.0	[10]
**$CsPbI_3$**	2.5 eV	15.0	[5]
**$(CH_3NH_3)_3Bi_2I_9$**	2.1 eV	13.2	[11]
**$FAPbI_3$**	1.49 eV	2.1	[12]
**$(C_4H_9NH_3)_2PbI_4$**	2.28 eV	27.5	[13]
**$(C_6H_5CH_2NH_3)_2PbI_4$**	2.19 eV	20.1	[14]

Table S1. The bandgap at ambient conditions and the pressures reaching the S-Q limit bandgap of halide perovskites ever reported.

Halide Perovskites	Metallization Pressure	Refs.
**$(NH_4)_2PtI_6$**	14.2 GPa	This work
**$Cs_3Bi_2I_9$**	28 GPa	[9]
**$Cs_3Sb_2I_9$**	44.3 GPa	[10]
**$CsPbI_3$**	39.3 GPa	[5]
**$(CH_3NH_3)_3Bi_2I_9$**	60 GPa	[11]
**$(CH_3NH_3)PbI_3$**	60 GPa	[15]

Table S2. The metallization pressures of halide perovskites ever reported.

$(NH_4)_2PtI_6$

	1 atm	1.5 GPa	6.2 GPa
**temperature/K**	293(2)	293(2)	293(2)
**crystal system**	cubic	tetragonal	monoclinic
**space group**	*Fm*-3*m*	*P*4/*mnc*	*C*2/*c*
**a / Å**	11.1593	7.6192	7.1182
**b / Å**	11.1593	7.6192	7.4208
**c / Å**	11.1593	11.3565	11.0953
**α/°**	90	90	90
**β /°**	90	90	89.0728
**γ /°**	90	90	90
**Volume / Å^3**	1389.6673	659.2669	586.0074
**θ range /°**	5-20	5-20	5-20
**Wavelength / Å**	0.6199	0.6199	0.6199
**R_{wp} / %**	0.95	1.13	0.39
**R_p / %**	0.52	0.74	0.27

Table S3. Refinement lattice parameters and refinement statistics at ambient pressure, 1.5 GPa and 6.2 GPa for $(NH_4)_2PtI_6$ crystal.

	Pressure (GPa)	a (Å)	b (Å)	c (Å)	V (Å^3)
**Cubic (*Fm*-3*m*)**	**0**	11.1593	11.1593	11.1593	1389.6673
	**0.3**	11.1356	11.1356	11.1356	1380.8321
**Tetragonal (*P*4/*mnc*)**	**0.5**	7.8062	7.8062	11.1903	681.9006
	**0.8**	7.71904	7.71904	11.30523	673.60545
	**1.5**	7.61921	7.61921	11.35651	659.2711
	**2.7**	7.4807	7.4807	11.3141	633.1469
	**3.6**	7.4072	7.4072	11.2677	618.2205
**Monoclinic (*C*2/*c*)**	**4.7**	7.2339	7.5504	11.1972	611.5136
	**6.2**	7.1182	7.4208	11.0953	586.0074
	**7.3**	7.0623	7.3796	11.0464	575.6186
	**9.1**	6.9333	7.3324	10.9650	557.2153
	**10.0**	6.9021	7.2990	10.9331	550.5525
	**11.8**	6.8913	7.1391	10.8710	534.7035
	**14.0**	6.8278	7.0284	10.7915	517.7687
	**16.8**	6.7374	6.9344	10.6869	499.1944
	**18.5**	6.6641	6.7887	10.6557	481.3593
	**20.4**	6.6355	6.7736	10.6324	476.9224

Table S4. The lattice parameters and unit cell volume of $(NH_4)_2PtI_6$ at different pressures.

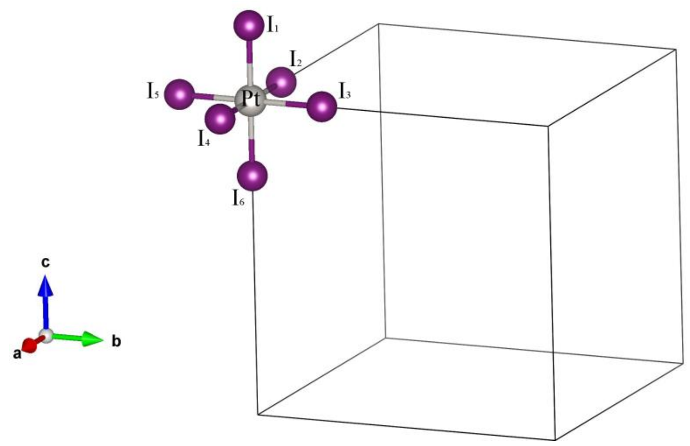

Phase	Cubic	Tetragonal	Monoclinic
**(GPa)**	0.0	1.5	6.2
**Pt-I$_1$**	2.6693(0)	2.6063(2)	2.5463(7)
**Pt-I$_2$**	2.6693(0)	2.6677(3)	2.5280(1)
**Pt-I$_3$**	2.6693(0)	2.6677(3)	2.5635(4)
**Pt-I$_4$**	2.6693(0)	2.6677(3)	2.5280(1)
**Pt-I$_5$**	2.6693(0)	2.6677(3)	2.5635(4)
**Pt-I$_6$**	2.6693(0)	2.6063(2)	2.5463(7)
**I$_1$-Pt-I$_2$**	90.000(0)	90.000(0)	90.746(9)
**I$_1$-Pt-I$_3$**	90.000(0)	90.000(0)	89.480(4)
**I$_2$-Pt-I$_3$**	90.000(0)	90.000(0)	87.753(3)
**I$_4$-Pt-I$_5$**	90.000(0)	90.000(0)	87.753(3)
**I$_4$-Pt-I$_6$**	90.000(0)	90.000(0)	90.746(9)
**I$_5$-Pt-I$_6$**	90.000(0)	90.000(0)	89.480(4)

Table S5. Pt-I bond lengths and I-Pt-I bond angles of $(NH_4)_2PtI_6$ under various pressures. The illustration shows the positions of Pt and I atoms.

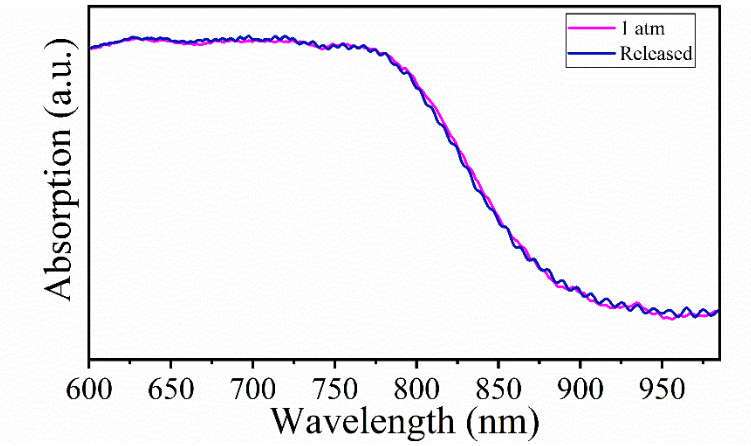

Figure S1. The absorption spectra of the $(NH_4)_2PtI_6$ crystal before and after the high-pressure treatments.

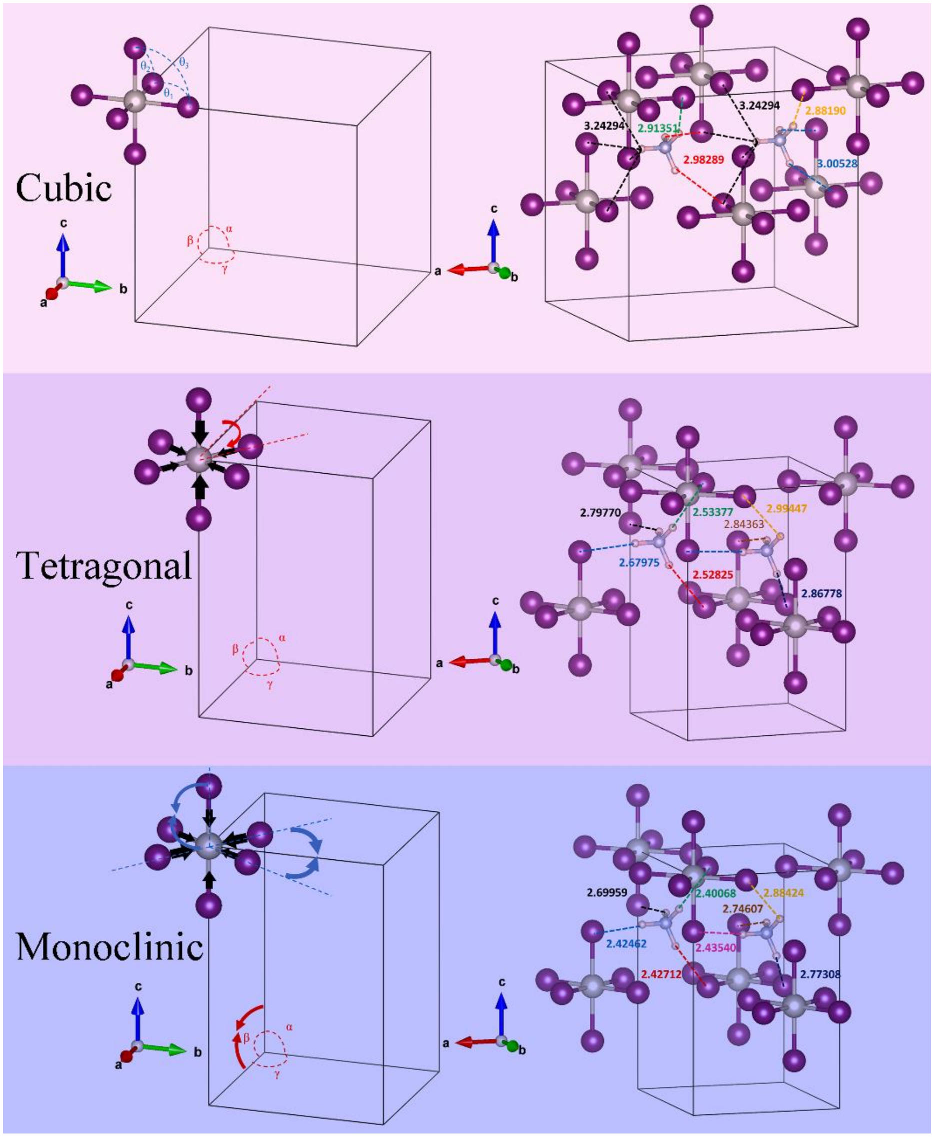

Figure S2. Schematic illustrations with respect to polyhedral views of crystal structures of $(NH_4)_2PtI_6$ perovskites under high pressure.

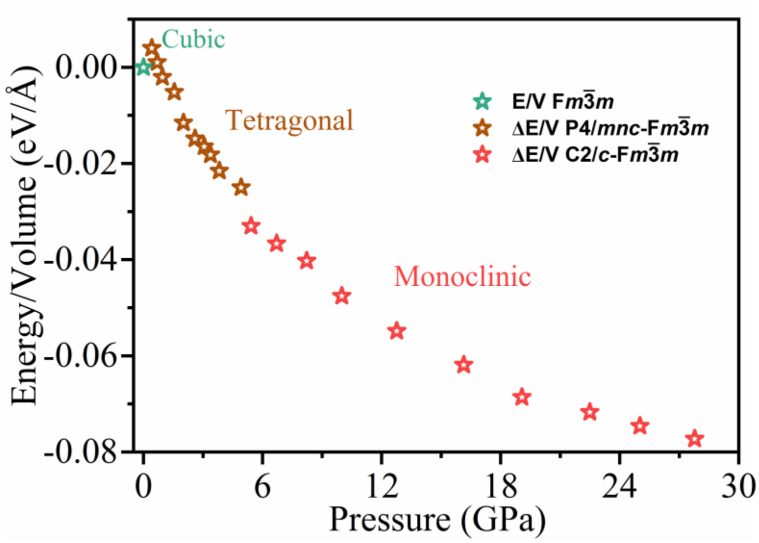

Figure S3. The calculated energy difference per unit volume of difference phases under the pressure.

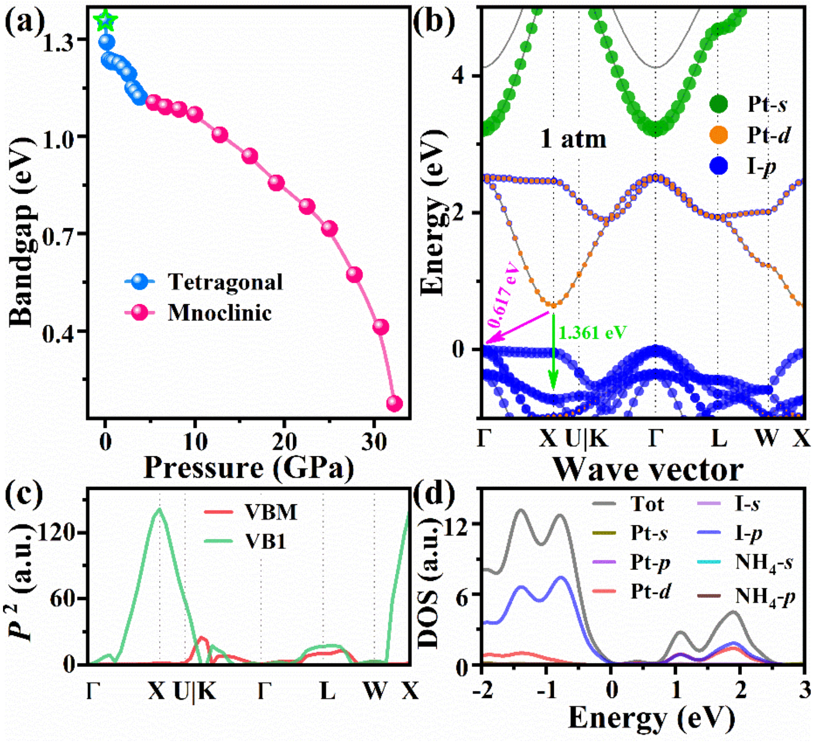

Figure S4. a) DFT-HSE06 calculated bandgap of $(NH_4)_2PtI_6$ under various pressures. The green-star shows the bandgap of cubic phase at ambient pressure. b) The projected band structure for the Pt and I atoms under ambient conditions. c) The calculated square of the transition dipole moment (P^2) along the high symmetry lines for VBM and VB1. d) Total and projected density of states (DOS and PDOS) in cubic phase under 1 atm of $(NH_4)_2PtI_6$.

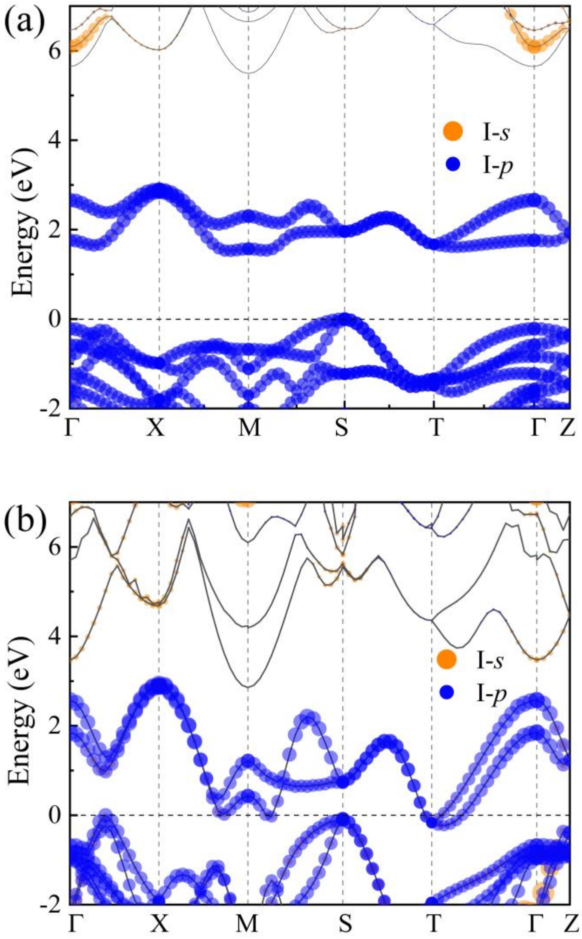

Figure S5. The projected band structures of bulk I (*Cmca*) at a) 1 atm, b) 16.0 GPa, respectively.

References

[1] a) G. Kresse, J. Furthmüller, *Phys. Rev. B* **1996**, *54* (16), 11169; b) *Comput. Mater. Sci.* **1996**, *6* (1), 15.

[2] a) P. E. Blöchl, *Phys. Rev. B* **1994**, *50* (24), 17953; b) J. P. Perdew, K. Burke, M. Ernzerhof, *Phys. Rev. Lett.* **1996**, *77* (18), 3865.

[3] S. Grimme, *J. Comput. Chem.* **2006**, *27* (15), 1787.

[4] L. Stixrude, C. Lithgow-Bertelloni, B. Kiefer, P. Fumagalli, *Phys. Rev. B* 2007, **75**, 024108.

[5] Y. Liang, X. Huang, Y. Huang, X. Wang, F. Li, Y. Wang, F. Tian, B. Liu, Z. X. Shen, T. Cui, *Adv. Sci.* **2019**, 6(14), 1900399.

[6] C. Gao, R. Li, Y. Li, R. Wang, M. Wang, Z. Gan, L. Bai, Y. Liu, K. Zhao, S. F. Liu, Y. Cheng, W. Huang, *J. Phys. Chem. Lett.* **2019**, 10(19), 5687.

[7] A. V. Krukau, O. A. Vydrov, A. F. Izmaylov, G. E. Scuseria, *J. Chem. Phys* **2006**, *125* (22), 224106.

[8] V. Wang, N. Xu, J.-C. Liu, G. Tang, W.-T. Geng, *Comput. Phys. Commun.* **2021**, *267*, 108033.

[9] L. Zhang, C. Liu, L. Wang, C. Liu, K. Wang, B. Zou, *J. Phys. Chem. Lett.* **2018**, *57* (35), 11213.

[10] L. Wu, Z. Dong, L. Zhang, C. Liu, K. Wang, B. Zou, *ChemSusChem* **2019**, *12* (17), 3971.

[11] L. Zhang, C. Liu, Y. Lin, K. Wang, F. Ke, C. Liu, W. L. Mao, B. Zou, *J. Phys. Chem. Lett.* **2019**, *10* (8), 1676.

[12] G. Liu, L. Kong, J. Gong, W. Yang, H.-k. Mao, Q. Hu, Z. Liu, R. D. Schaller, D. Zhang, T. Xu, *Adv. Funct. Mater.* **2017**, *27* (3), 1604208.

[13] Y. Yuan, X.-F. Liu, X. Ma, X. Wang, X. Li, J. Xiao, X. Li, H.-L. Zhang, L. Wang, *Adv, Sci.* **2019**, *6* (15), 1900240.

[14] C. Tian, Y. Liang, W. Chen, Y. Huang, X. Huang, F. Tian, X. Yang, *Phys. Chem. Chem. Phys.* **2020**, *22* (4), 1841.

[15] A. Jaffe, Y. Lin, W. L. Mao, H. I. Karunadasa, *J. Am. Chem. Soc.* **2017**, *139* (12), 4330.
